# Co-extinction in a host-parasite network: identifying key hosts for network stability

**DOI:** 10.1038/srep13185

**Published:** 2015-08-17

**Authors:** Tad Dallas, Emily Cornelius

**Affiliations:** 1 Odum School of Ecology, University of Georgia, Athens, GA 30606 – 4288; 2 Forest and Wildlife Ecology, University of Wisconsin, Madison, WI 53706-1799.

## Abstract

Parasites comprise a substantial portion of total biodiversity. Ultimately, this means that host extinction could result in many secondary extinctions of obligate parasites and potentially alter host-parasite network structure. Here, we examined a highly resolved fish-parasite network to determine key hosts responsible for maintaining parasite diversity and network structure (quantified here as nestedness and modularity). We evaluated four possible host extinction orders and compared the resulting co-extinction dynamics to random extinction simulations; including host removal based on estimated extinction risk, parasite species richness and host level contributions to nestedness and modularity. We found that all extinction orders, except the one based on realistic extinction risk, resulted in faster declines in parasite diversity and network structure relative to random biodiversity loss. Further, we determined species-level contributions to network structure were best predicted by parasite species richness and host family. Taken together, we demonstrate that a small proportion of hosts contribute substantially to network structure and that removal of these hosts results in rapid declines in parasite diversity and network structure. As network stability can potentially be inferred through measures of network structure, our findings may provide insight into species traits that confer stability.

Species extinctions can alter community dynamics^1^, the transfer of energy through ecosystems, and may also result in secondary extinctions of obligate species^2^,^3^,^4^,^5^. As obligate species, parasites represent a hidden portion of biodiversity, comprising around half of the planet’s total biodiversity, and are important indicators of ecosystem health^3^,^6^. Perturbations, such as habitat loss and climate change, may endanger free-living species and their resident parasites. Given the diversity of parasite communities occupying hosts, the extinction of a host species could result in many cryptic, secondary extinctions of specialist parasites. Further, the loss of a host species and the corresponding secondary extinctions have a potential to impact the structure of the host-parasite network, which could have large destabilizing effects and perhaps result in further species loss^7^.

Despite the potential importance of host species identity, traits, and interaction patterns to secondary extinction dynamics^1^ and community stability, few studies have examined network structure or stability from a trait-based perspective, though this has been identified as a future research need^5^. For instance, many co-extinction investigations in food webs remove species based on their number of interactions (i.e. degree)^8^,^9^. However, the order which hosts become locally extinct from a network is nontrivial, as different extinction scenarios will result in vastly different outcomes^10^,^11^. Recent efforts have focused on refining the traditional node removal strategy by incorporating elements of species biology, such as host range size, trophic level, and estimated extinction risk^3^,^12^,^13^. However, much of the previous work on co-extinction dynamics has been in food webs, which are unipartite networks, and differ fundamentally from bipartite networks, in which two classes of organisms interact with each other, but not among nodes in the same class. Finally, most studies to date have quantified extinction cascades in ecological networks by losses in biodiversity, without assessing changes to network structure. Here, we examine both losses to biodiversity (secondary extinctions) and changes to network structure with the removal of host species from a network.

Network structure is commonly quantified using topological network measures to describe the tendency for interactions in a network to be organized in a particular manner. These metrics describing network structure have often been related to measures of network stability, such as structural stability^14^. Understanding how network structure changes with host species loss is an interesting unexplored research question. We focus on two measures of network structure which have been previously related to network stability; nestedness and modularity^14^,^15^,^16^,^17^,^18^,^19^. Nested networks are observed when nodes with few links form subsets of the links observed for higher degree nodes^16^,^18^. For instance, in the context of host-parasite networks, hosts with species-rich parasite communities would contain all parasites found in hosts with sparser parasite communities. Modularity, on the other hand, is a measure of community formation^20^,^21^,^22^ and is the tendency for groups of nodes to have many interactions among them and sparse connections to other groups of highly-connected nodes^23^. We expect based on previous studies that nestedness and modularity will have opposite influences on network stability, as Thébault and Fontaine (2010)^17^ previously suggested that trophic network stability was related to modularity, while nestedness actually destabilized trophic networks.

Currently, co-extinction studies tend to measure the effects of an extinction event on the diversity of the network^2^,^3^,^13^, while studies of network structure tend to ignore the possibility of host extinction and focus on the role of particular hosts to networks structure^3^,^9^,^18^. The natural synthesis of these two approaches can provide predictions on what happens to network structure when species are systematically lost from the network. Here, we combine both approaches, using data from a Neotropical fish-parasite network^24^, to distinguish hosts contributing differentially to network structure, traits associated with these key hosts, and the resulting effect of removing these hosts relative to random extinction simulations. We provide evidence that 1) a small proportion of key hosts are responsible for maintaining network structure (i.e. nestedness and modularity), 2) parasite diversity and host family are important predictors of species level contributions to network structure, and 3) simulated extinctions of hosts with high parasite richness, or large contributions to network structure (nestedness and modularity), tend to result in more precipitous declines in parasite diversity and network structure relative to random simulations.

## Materials and Methods

### Study system

The current study utilizes data from a Long Term Ecological Research study on the fish and parasite communities of the Upper Paraná River floodplain, a 230 km long dam-free portion of the Paraná River in southern Brazil. Fish were sampled quarterly for eight years (2000–2008) by using both active and passive sampling techniques, resulting in a total of 4,875 fish captures of 72 species selected for parasitological analysis, Native species (n = 49) outnumbered non-native species (n = 23) of those fish species chosen for parasitological analyses. Parasites (n = 324) were identified to the best possible resolution, which resulted in 140 parasites identified to the species level, 120 to genus, and 64 to family^24^. The network is significantly nested and modular^24^, and has low connectance (*C* = 0.022), similar to other fish-parasite networks^25^. Details for the sampling protocols^26^ and parasite examination^27^,^28^ are provided elsewhere. Calculations of network structure metrics were based on the host-parasite interaction matrix provided by Lima *et al*. (2012)^24^.

### Fish host trait variables selected

Data on fish traits (Table 1) were obtained from FishBase [Froese, R. and D. Pauly, www.FishBase.org, accessed 08/2014] a comprehensive fish life history trait database accessible in *R* using *rfishbase*^29^, and based on information provided in Lima *et al*. (2012)^24^. The importance of certain fish species in the network may relate to specific host traits. For instance, body size and range size are often correlated, and larger ranged hosts typically harbor higher parasite diversity. Loss of these hosts could have a larger negative impact on network structure. However, we did not exclude variables for which we had data on, as there was no *a priori* reason to exclude data, and our analysis method was insensitive to issues of collinearity and overparameterization. Parasite species richness was quantified as the number of parasite species found on a given host species. Measures obtained from FishBase included host extinction risk (“vulnerability”)^30^, resilience from a perturbation^31^, phylogenetic diversity^32^, maximum weight, body size, length at maturity, and host family. Vulnerability is a predicted measure of extinction risk based on host life history information. Resilience is a measure that incorporates host demographic information (carrying capacity, fecundity, etc.) to classify the likelihood that a fish population will recover quickly from a perturbation. Phylogenetic diversity is reported in FishBase and based on Faith *et al*. (2004)^32^. The specific measure used, PD_50_, represents the expected loss in phylogenetic diversity under species extinction, assuming that all other species have equiprobable extinction risk. Measure of sampling effort, fish abundance, and biomass were obtained from Lima *et al*. 2012^24^. Host species “status” (i.e., native or non-native) was compiled by Lima *et al*. (2012)^24^, though the original evaluation was performed by Julio *et al*. (2009)^33^.

### Host species-level contribution to nestedness and modularity

We quantified nestedness using a metric based on overlap and decreasing matrix fill (NODF)^34^. Nestedness contribution (C_i_), a measure of the importance of a single node to overall network structure, was calculated following Saavedra (2011)^18^, in which the interactions of a single host species were iteratively randomized, with the resulting simulated NODF values compared to the observed nestedness of the empirical network. Modularity contribution (Q_i_) was calculated in the same way, but using Barber’s *Q* statistic^20^. Both measures were calculated only for the complete network. It is likely that species contribution to nestedness and modularity changes as the network disassembles. However, identifying nestedness and modularity contributions via dynamic updating makes the measures state-dependent, in such that the relative impact of a species removal in the full network may be greatly altered after a certain number of nodes have been removed. As a result, our modularity contribution values do not contain information on how the network will respond when host species are lost from the network. Values of C_i_ or Q_i_ close to zero indicate little or null impact of that host on network structure, while positive (negative) values are indicative of hosts that enhance (reduce) nestedness or modularity.

We used three randomization algorithms ranging from *a)* each interaction equiprobable *b)* probability of interaction determined by parasite host breadth and *c)* probability of interaction determined by average of host and parasite occurrence probabilities (supplementary equation S1; Saavedra 2011^18^). All null models gave similar results, so we report parasite host breadth to determine occurrence probabilities herein (*b* from above), and relationships between measures obtained from the different null models are presented as supplementary material (Figs S1 & S2).

### Traits associated with host nestedness contribution

In order to assess the potential relationship between host traits and their corresponding contributions to network structure (i.e. C_i_ and Q_i_), we used regression tree analysis. Regression trees are a useful tool for the identification of the most important predictor variables, and have been used extensively in ecological applications^35^,^36^,^37^,^38^. Regression tree analysis aims to predict a response variable based on several input variables, in which input variables represent splits in the tree. We applied an iterative process called “boosting”, where we formed many regression trees, and then combined to improve predictive power. The final boosted regression tree (BRT) model was cross-validated using ten-fold cross validation and permutation tests to determine the optimal number of trees. Variables included in the BRT analysis are provided in Table 1. The relative contribution (RC) of predictor variables was determined through permutation tests, in which each predictor variable was permuted randomly, and the importance of the predictor variable was quantified by the subsequent reduction in model performance. Relative contribution estimates are based on the number of times a given predictor variable is selected for splitting, weighted by the degree the split improves model performance. This metric is then averaged across all trees built in the model, and scaled between 0 (no contribution) to 100 (high contribution).

### Host removal simulations

In order to assess the impact of host extinction on network structure and parasite diversity, we sequentially removed hosts according to four potential extinction orders. We removed hosts from the network in order from highest to lowest host extinction risk (as estimated by Cheung *et al*. 2005)^30^, parasite species richness, modularity contribution, and nestedness contribution. Many of these variables are related, which also makes the extinction orders related (see Figure S4 for rank correlations between all species removal scenarios).

After each host removal, nestedness (NODF), modularity (Q), and parasite diversity (PSR; total number of parasite species remaining) were determined. Hosts were removed in random order for 1000 simulations to provide appropriate bounds of expected trajectories for the decay of nestedness, modularity, and parasite diversity. To measure the magnitude of the effect of each of the four species removal scenarios, we compared the decay curves of our targeted removal scenarios to a null expectation generated by 1000 random extinction simulations. From these simulations, we quantified the divergence of our targeted extinction scenarios to random simulations. To do this, we first plotted the decay in parasite diversity, modularity, or nestedness as a function of the number of hosts removed for each of our targeted and random simulations. Then we quantified the decay area (*DA*) between the curve generated from a targeted extinction scenario and the mean curve generated from random extinction simulations in terms of parasite diversity (*DA*_*PSR*_), nestedness (*DA*_*C*_), and modularity (*DA*_*Q*_). This measure is almost identical to how Evans *et al*. (2013)^39^ defined robustness, except they considered robustness the area under the decay curve to the origin instead of to a mean random simulated extinction scenario.

We also calculated *DA* values for each random simulation to the mean random simulation, thus creating a distribution of possible decay area values obtained from completely random extinction simulations. We treated this area as an estimate of the magnitude of difference between a particular host extinction scenario and random extinction simulations, which allows us to compare random extinction scenarios to our targeted extinction scenarios using *z*-tests. We performed all analyses in the R Statistical Computing Environment using functions from the *vegan* package for the analysis of nestedness [Oksanen *et al*. 2012], *gbm* for the boosted regression tree analysis [Ridgeway *et al*. 2013], and *flux* for AUC determination [Jurasinski *et al*. 2014].

### Data accessibility

Host-parasite interaction matrices are provided in the supplementary material of Lima *et al.* (2012). Fish host traits were obtained from FishBase (Froese and Pauly 2012) and from Agostinho *et al.* (2004).

## Results

### Host traits associated with network structure

Hosts contributed differentially to network properties, resulting in many hosts having a negligible effect on nestedness or modularity, while a small number of hosts had large positive effects on network structure measures. Contrary to the idea that modularity and nestedness have opposing influences on network structure (i.e. should be negatively correlated), we found a significant positive relationship between the two measures (r_s_ = 0.485, *p*-value < 0.0001).

**Figure 1:**
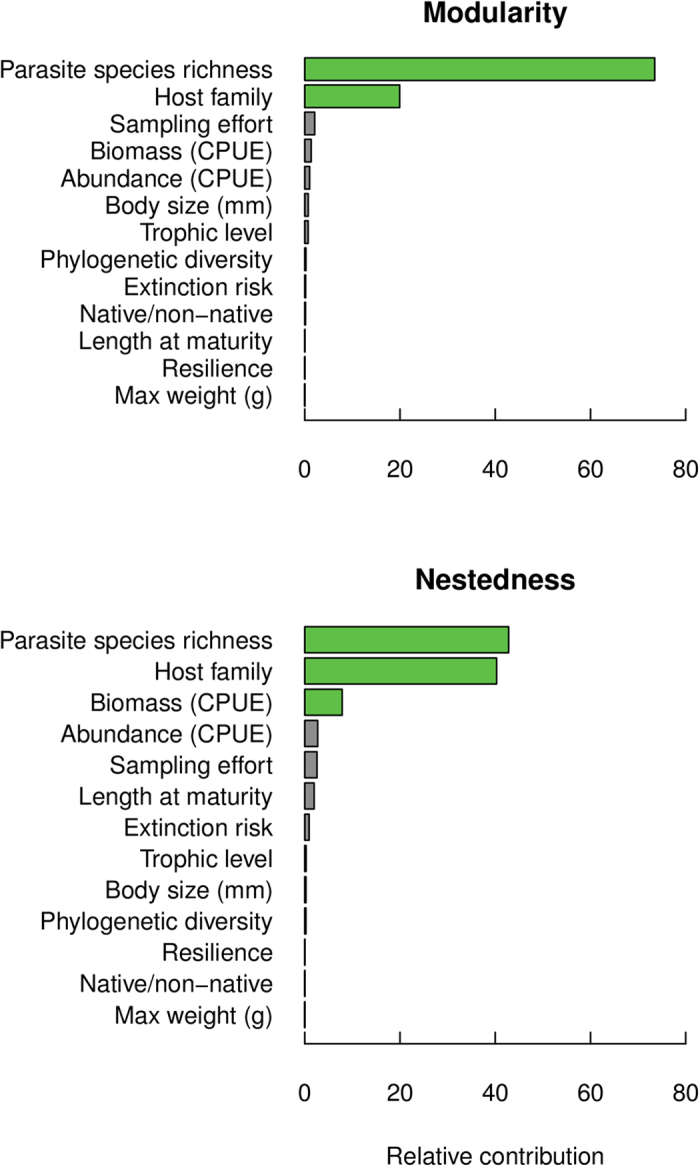
Species contributions to nestednesss and modularity as functions of life history traits. Boosted regression models for modularity (top) and nestedness (bottom) contributions of host species, with green bars indicating fish species traits associated with their contribution to either measure of network structure (relative contribution values greater than 5%).

Host-specific contributions to nestedness and modularity were most associated with parasite species richness based on the relative contribution (RC) values obtained from the boosted regression tree analysis (Fig. 1). Host family was the second-most important variable to both nestedness contributions (RC = 40.28) and modularity contributions (RC = 19.94) of host species, indicating a potential role of host phylogeny in determining the host species relative impact on network structure. The biomass of fish captured per host species had a marginal influence on host-specific contributions to nestedness. The remaining variables contributed little, each having a relative contribution value of less than 5 units (out of 100). To examine the predictive accuracy of our models, we regressed model predictions against actual values of nestedness and modularity contribution. Regression plots of model predicted versus actual values for nestedness and modularity contribution are provided in the Supplementary Materials (Figure S3). Root mean squared error is another commonly used scale-dependent measure of predictive accuracy. Nestedness contribution (RMSE = 1.06) and modularity contribution (RMSE = 1.05) both had relatively low RMSE values, slightly over one unit difference between predicted and actual values. Both biomass and abundance are variables defined in terms of catch per unit effort, which makes both variables closely related to the dominance of a species in a community, and to sampling effort, as larger values would indicate species more frequently examined for parasites. However, sampling effort was not a strong contributor to either measure of network structure (C_i_ and Q_i_), suggesting the importance of biomass to C_i_ could relate more to host biology and community composition than sampling effort.

### Effect of nonrandom host extinctions on secondary extinctions

**Figure 2:**
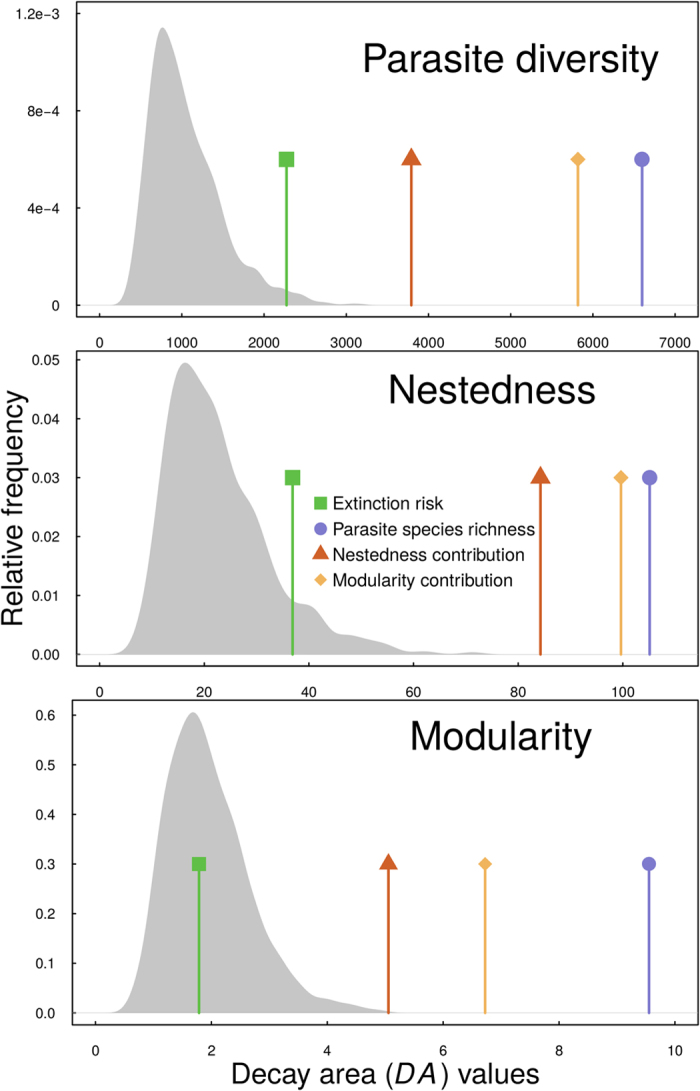
Histograms of the distribution of decay area (*DA*) values of parasite diversity nestedness and modularity for random simulations, with points indicating the *DA* values for each of the four ordered extinction scenarios.

**Figure 3:**
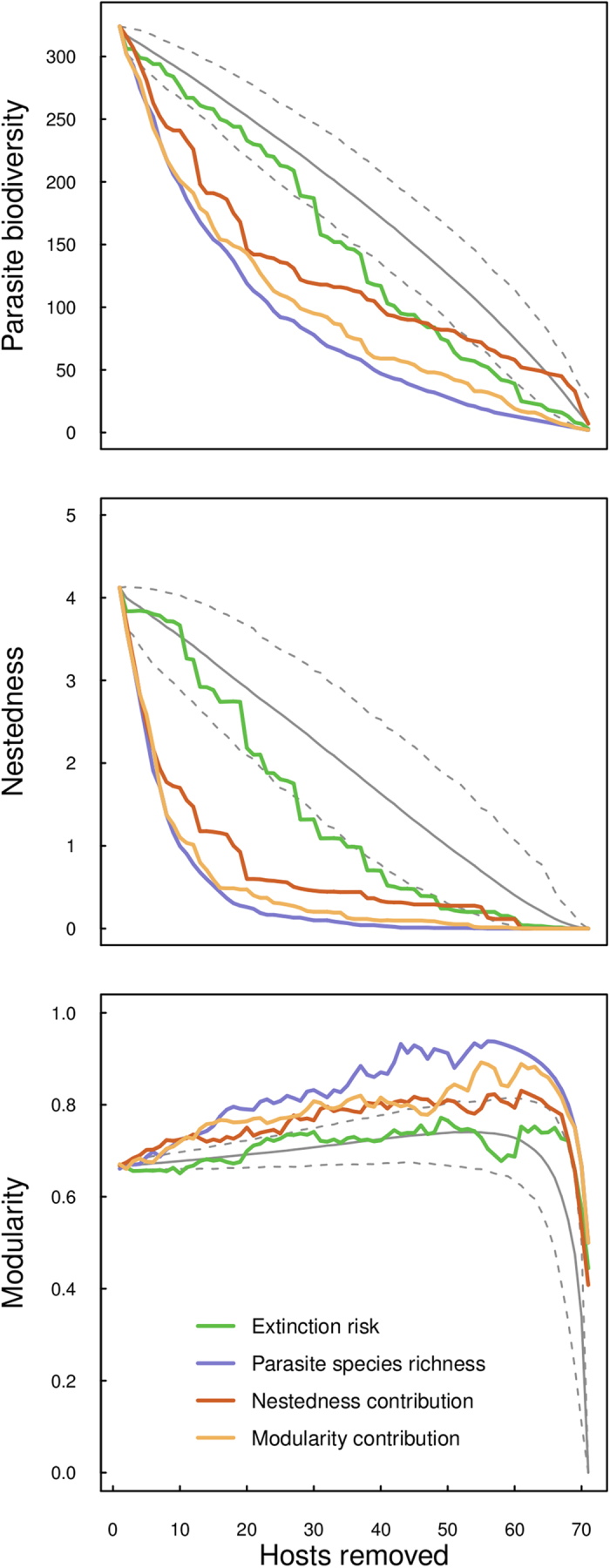
Simulated trajectories examining the decay in parasite diversity (top), nestedness (middle), and modularity (bottom) as a result of simulated sequential host removals. Host removal based on parasite species richness (purple) resulted in the fastest declines of network metrics examined, followed closely by host removal based on modularity contribution (yellow) and nestedness contribution (red). Host removal by estimated extinction risk (green) did not differ from random extinction in terms of nestedness and modularity losses, but parasite diversity did decline faster relative to random simulations.

Sequential host removal scenarios based on extinction risk, parasite species richness, nestedness and modularity contributions resulted in significantly larger decay areas (*DA*) in parasite diversity(*DA*_*PSR*_), nestedness (*DA*_*C*_), and modularity (*DA*_*Q*_) (Figs 2 and 3) compared to random simulations. The exception to this was host removal based on estimated extinction risk, which resulted in significantly larger decay area when looking at parasite diversity, but not nestedness or modularity. In other words, parasite diversity, but not nestedness or modularity, declined at a faster rate when hosts were sequentially removed based on their estimated extinction risk (Table 2).

Host removal based on parasite species richness values resulted in larger decay area values than removal of hosts based on their estimated nestedness and modularity contribution (Table 2 and Fig. 3), suggesting both a strong link between parasite species richness and species level contributions to network structure (see Supplementary Material for rank correlation matrix) and also that nestedness and modularity contributions may change markedly as hosts are removed from the network. In other words, a policy that re-calculates nestedness and modularity contribution after each host removal may converge upon on a similar or better host removal strategy than removal based on parasite species richness.

Another interesting result is what happens to modularity values with any (random or non-random) host removal. Modularity values actually increase until a critical point, after which they go to zero. This is likely a result of the modularity calculation itself. We demonstrate this in the Supplementary Materials (Figure S6) by numerically simulating sequential host removal in randomly constructed bipartite networks. Understanding if this increase in modularity is a statistical artifact, or an important component of complex networks is an area for further research.

There are many host removal strategies not examined here. For instance, host extinction may be related to their native or non-native status, or their abundance within the community. While these analyses may be outside of the scope of the current manuscript, we have examined both in the Supplementary Materials (Figure S5 in particular).

## Discussion

Declining host biodiversity could result in secondary extinctions of obligate parasites, especially parasites endemic to a small subset of host species. These secondary extinctions, in turn, may result in further extinctions as demonstrated recently in a host-parasitoid system^40^. Coextinction may destabilize networks^41^, resulting in faster losses in biodiversity and network structure than expected under random extinction scenarios. Here, we find that the differential impact a host has on network structure and parasite diversity can be partially explained by host traits. First, we found that hosts contributing strongly to nestedness also contributed strongly to modularity, partially reconciling two opposing network properties related to network structure^42^, though more research is necessary to determine the true relationship between these network properties. Secondly, we found that both network structure measures were related to host family affiliation and parasite community richness, suggesting that host phylogeny and the number of interactions a host has in the network play a role in determining network structure. Also, fish biomass was marginally important in predicting nestedness contributions, which may relate to biased sampling, or to host body size, as fish biomass combines aspects of individual fish mass and relative abundance. Taken together, these findings suggest that species roles in antagonistic networks may be phylogenetically conserved or influenced by host traits (e.g. body mass). Lastly, we found that random extinction simulations may underestimate the rate of decline in parasite diversity when compared to extinction based on realistic extinction risk measures, and that extinction orders based on parasite species richness, and network structure contribution measures tended to result in much faster declines in parasite diversity and nestedess relative to random extinction simulations.

Previous studies have argued that topological measures of network structure may confer stability^14^,^17^. For instance, nestedness^43^ and modularity^17^ have both been related to network persistence (proportion of surviving species after recovery from perturbation), and resilience (time until community returns to equilibrium after perturbation). Further, resilience, robustness and structural stability (all slightly different components of stability) have previously been related to nestedness^14^. These topological measures are appealing proxies for network stability as they are easy to measure and do not require time-series data to calculate. Previous studies have acknowledged that some species are more important for network stability than others^8^,^9^,^44^. Our decay area (*DA*) statistic can be considered a measure of network robustness^39^, thus relating topological properties of networks (nestedness, and modularity) to dynamical properties (stability).

While the importance of host family to species level contributions to nestedness and modularity could be viewed in an evolutionary context, it is potentially a result of the variation in parasite species richness seen in the fish taxa examined. For example, the family *Anostomidae* (specifically four native *Leporinus* species) had the highest positive values of C_i_ and Q_i_ and also the highest parasite species richness relative to other fish taxa. While the importance of host family may be conflated with parasite species richness, it is also possible that host family represents a coarse measure of phylogenetic relatedness, such that host family can be considered a proxy for evolutionary relatedness. Alternatively, this could simply be a variable related to unmeasured trait variation. Rank correlations between extinction scenarios show support for the relationship between species contributions to network structure and parasite species richness (Figure S4). Overall, this suggests that parasite species richness may be the most important determinant of network structure and parasite diversity, even when compared to metrics that calculate species-level contributions to nestedness and modularity. There are a number of possible explanations for this.

First, nestedness and modularity contribution metrics were not dynamically updated as species were removed. This was intentional, as the goal of this study was to determine if a snapshot of a full network could capture node importance as the network disassembles. This result makes intuitive sense, as the removal of host species with species-rich parasite communities may be more likely to result in co-extinction of parasite species. This finding, despite its appealing simplicity may not be true for some networks, as other aspects of network structure, host biology and community dynamics may also be important for network stability^45^. Second, it may be the case that nestedness and modularity contribution measures simply cannot capture a host species true contribution to network structure, such that more simple metrics like node degree (i.e., parasite species richness) may outperform more complicated metrics. Lastly, the calculation of nestedness and modularity contributions is based on null model randomizations of each host in the network. That is, nestedness and modularity contribution measures are based on the idea that the host is still present, but the interactions of that host are randomized. Statistically, this approach makes sense, since some network structure metrics are sensitive to matrix size or fill. However, this formulation is conceptually different from a host removal, which may explain why removal strategies based on nestedness and modularity contributions did not result in the fastest declines in network structure.

Through simulated sequential host extinctions, we found that parasite diversity declined at a slightly faster rate when extinction order was based on host extinction risk than compared to random extinction order, but only after around 40% of hosts were already lost from the system. This suggests that the loss of host biodiversity based on realistic estimates of extinction risk may result in a greater loss of parasite diversity through secondary extinctions than is currently expected, but only in highly perturbed systems subject to large losses in host diversity. This result is comparable to Davies and Yessoufou (2013)^46^, in which they found that phylogenetically clustered extinctions result in disproportionate loss, compared to random extinction scenarios.

While parasite diversity and nestedness declined precipitously in our simulations, modularity actually increased slowly in our extinction scenarios relative to random extinction simulations. This finding may be a function of removing hosts with high parasite species richness as this may fragment the network into small groups of specialist parasites, which would increase modularity. In a limiting case, a host-parasite network in which each host is parasitized by a single host-specific parasite would be perfectly modular. However, modularity increased even in random extinction simulations, suggesting one of two things; either modularity statistics are biased by matrix size or host removal from a network may actually increase modularity until a critical point is reached before the network collapses entirely. We address the relationship between matrix size and modularity in the Supplementary Materials (Figure S6).

Biodiversity loss is a pressing concern, with recent focus on the impact of host extinctions on secondary extinctions of obligate parasites^6^ and the importance of parasites as indicators of ecosystem health^47^,^48^. A recent investigation into the shape of the relationship between free-living species richness and parasite richness under sequential host removal simulations resulted in a myriad of possible trajectories for the decay of parasite diversity^6^. While we acknowledge that host diversity loss may influence parasite diversity idiosyncratically, our simulations find a consistently greater loss in both network stability and parasite diversity relative to random simulations. Understanding the network properties that alter decay trajectories (e.g. degree distribution) may provide a better understanding of the structure and stability of ecological networks^49^. Lastly, the integration of network structure measures into studies of network stability may yield insight into species-level characteristics that promote biodiversity and network structure.

## Additional Information

**How to cite this article**: Dallas, T. and Cornelius, E. Co-extinction in a host-parasite network: identifying key hosts for network stability. *Sci. Rep*. **5**, 13185; doi: 10.1038/srep13185 (2015).

## Supplementary Material

supplementary information

## Figures and Tables

**Table 1:** Fish life history traits (means and standard errors) used to train the boosted regression tree models.

Host trait	Units	Mean (sd)	n
Parasite species richness	Number of parasites per fish species	7.08 (8.21)	72
Host extinction risk	–	30.77 (17.58)	70
Body size	Maximum body size (mm)	354.87 (211.32)	72
Max weight	kg	9.11 (2.02)	25
Length at maturity	mm	49.50 (65.73)	43
Trophic level	–	3.08 (0.76)	70
Sampling effort	Number of fish analyzed for parasites	67.71 (92.28)	72
Abundance	CPUE (individuals/1000 m^2^ gill net)	27.25 (55.33)	69
Biomass	CPUE (kg/1000 m^2^ gill net)	3.74 (6.72)	69
Phylogenetic diversity	–	0.56 (0.13)	70
Resilience	Minimum population doubling time (years)	3.41 (3.16)	55
Status	Native or non-native	–	72

These outcomes related fish species life history traits to their contributions to network structure (nestedness and modularity). Descriptions of how fish life history metrics were assigned can be found in the materials and methods section.

**Table 2:** The effect of targeted extinction scenarios on the decay of parasite diversity, nestedness and modularity.

Removal order	Parasite diversity		Nestedness		Modularity	
	z	p	z	p	z	p
Extinction risk	2.81	**0.005**	−1.43	0.153	−0.24	0.81
Parasite species richness	12.73	**<0.0001**	8.37	**<0.0001**	10.63	**<0.0001**
Nestedness contribution	6.29	**<0.0001**	6.25	**<0.0001**	4.34	**<0.0001**
Modularity contribution	10.94	**<0.0001**	7.81	**<0.0001**	6.67	**<0.0001**

Decay area (*DA*) between targeted host removal orders were compared against *DA* values obtained by comparing random simulations to the mean decay trajectory.
