# Supplementary material for: Co-extinction in a host-parasite network: identifying key hosts for network stability
Source: Sci Rep. 2015 Aug 17;5:13185. doi: 10.1038/srep13185 (PMC4538395; doi:10.1038/srep13185)
Supplement: supplementary information [file srep13185-s1.doc]

**Co-extinction in a host-parasite network: identifying key hosts for network stability**

**Tad Dallas and Emily Cornelius**

**Supplementary material**

**Methods**

*Null model randomization*

In the current study, we used a total of 3 null models to allocate expected host-parasite interactions. The null model used in the main text ('null 0') filled in parasite occurrences on hosts based on the probability of that parasite utilizing other hosts. This null treats host as equal, but bases the probability of parasite occurrence on the number of other hosts that parasite species utilizes, thereby reducing the probability of specialist parasite occurrence. A more liberal null model, 'null 2', randomizes parasite occurrences on a host species with the same probability. The final null model examined is the most conservative randomization technique examined, with parasite occurrences based on the average of the relative interactions for hosts and parasites. More specifically, occurrence probabilities *p_ij_* are given by the formula provided in Saavedra (2011) (Equation S1). Each null model was run for 1000 simulations for each host in the network.

**Equation S1:** Null model used by Saavedra (2011) and Bascompte *et al.* (2003), corresponding to 'null 2' in our analysis, to determine the probability of interaction (*p_ij_* ) between host *i* (*k_i_* ) and parasite *j* (*k_j_* ), given the host community H and parasite community P. Where, *k_i_* and *k_j_* are the degree of species i and j, respectively, and H and P are the size of the host and parasite community, respectively.

**Predictive accuracy of boosted regression tree models**

We compared predicted values of nestedness and modularity contributions from our trained boosted regression tree models, and compared them to the empirical values (Figure S3). A linear model fit to predicted versus actual values is our measure of accuracy. Both nestedness contribution (adjusted R^2^ = 0.58) and modularity contribution (adjusted R^2^ = 0.77) were predicted with fairly good accuracy by our model trained with host traits.

**Host removal based on host abundance or origin**

In addition to the analyses in the main text, we determined the effect of removing host species based on their abundance (measured as catch per unit effort), and whether the host species was native or non-native. Hosts were removed by decreasing abundance, such that the most abundant host was the first to be lost from the system. This should result in a large immediate effect to network structure, especially given the common positive relationship between host abundance and parasite species richness. Host removal by abundance resulted in similar, though smaller, decay area (*DA*) values to removal based on nestedness contribution. Host removal by host status was more difficult, as there are a large number of possible ways to order 72 hosts based on a binary distinction. To address this, we created a number (n=100) potential extinction orders that removed all native hosts from the system first, followed by the removal of all non-native hosts (Figure S5). Removal based on host abundance resulted in faster declines in parasite diversity (*Z* = 4.81, *p* < 0.0001), nestedness (*Z* = 3.81, *p* < 0.0001), and modularity (*Z* = 4.79, *p* < 0.0001) relative to random simulations. However, when native hosts were lost first, there was not a large effect on parasite diversity (*Z* = 0.005, *p* = 0.996), nestedness (Z = 0.48, *p* = 0.631), or modularity (*Z* = 1.43, *p* = 0.153).

**Why does modularity increase with host removal?**

The increase in modularity with host removal was unexpected, as it would seem to indicate that networks become more structured, and potentially more stable (Thebault and Fontaine 2010) with host removal. We argue here that this increase in modularity is likely an effect of matrix size, and may be an issue with the calculation of the statistic itself, rather than a property of complex networks. To examine this, we simulated random (n=45) bipartite networks containing 50 hosts and 50 parasites. Occurrences were drawn as a binomial random variable with probability 0.3. Hosts were removed until 80% of host species were lost. Hosts were either removed randomly, or ordered based on parasite species richness (from largest to smallest). Before each host removal, we calculated modularity using two statistics; Barber's *Q* (Barber 2007), and QuaBiMo (Dormann and Straub 2013), as well as calculating mean number of parasites per host (i.e. parasite species richness), mean parasite host range (i.e. parasite specificity), and connectance (i.e. fraction of links realized out of all potential links). Modularity increased with both random and targeted extinctions, though other aggregate network properties changed (Figure S6). Specifically, mean parasite specificity decreased in both extinction scenarios, which is unsurprising since the mean number of hosts parasitized by a given parasite will shrink as the number of available hosts is reduced. Parasite species richness and connectance were only reduced in the targeted extinction simulations, whereas no pattern was observed in random extinction simulations. It is interesting to note that modularity increases sharper with host removal when hosts are removed based on their degree. This suggests that the rate of increase in modularity is determined by changes to link density, instead of simply a function of matrix size. Here, link density is defined as the average number of interactions per node, treating hosts and parasites as equivalent. However, we see from the impact of host removal on parasite species richness and parasite specificity that the resulting decrease in link density is driven nearly exclusively by reductions in the mean number of infected host species (mean parasite specificity). Taken together, this suggests modularity measures may be sensitive to matrix size (specifically the number of host species), and link density.

**References**

1. Barber, M. J. Modularity and community detection in bipartite networks. *Physical Review E*. 76, 066102 (2007).
2. Dormann, C. F., and R. Strauß. 2013. Detecting modules in quantitative bipartite networks: the QuaBiMo algorithm. *arXiv [q-bio.QM]* 1304.3218.


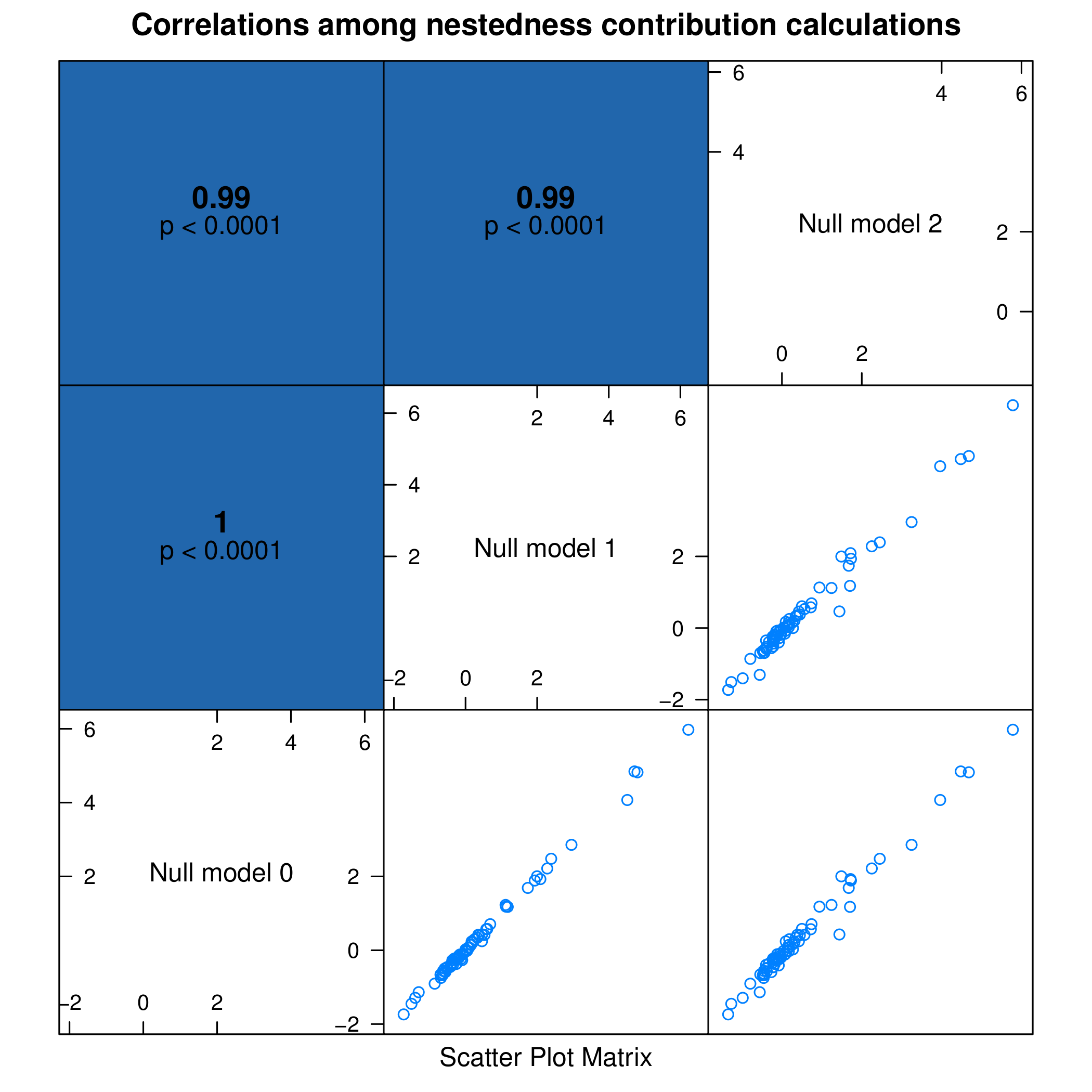


**Figure S1:** Correlations among estimates of host species contributions to nestedness, using three different null models. *Null model 0* is the most liberal, allowing cells in the interaction matrix to be filled randomly (e.g. only constraining the total number of interactions). *Null model 1* constrains the probability of an interaction between host and parasite by the number of hosts each parasite infests (e.g. column totals in the host-parasite matrix are constrained). *Null model 2* determines occurrence probabilities according to equation S1, which incorporates information on both row and column totals of the host-parasite matrix.


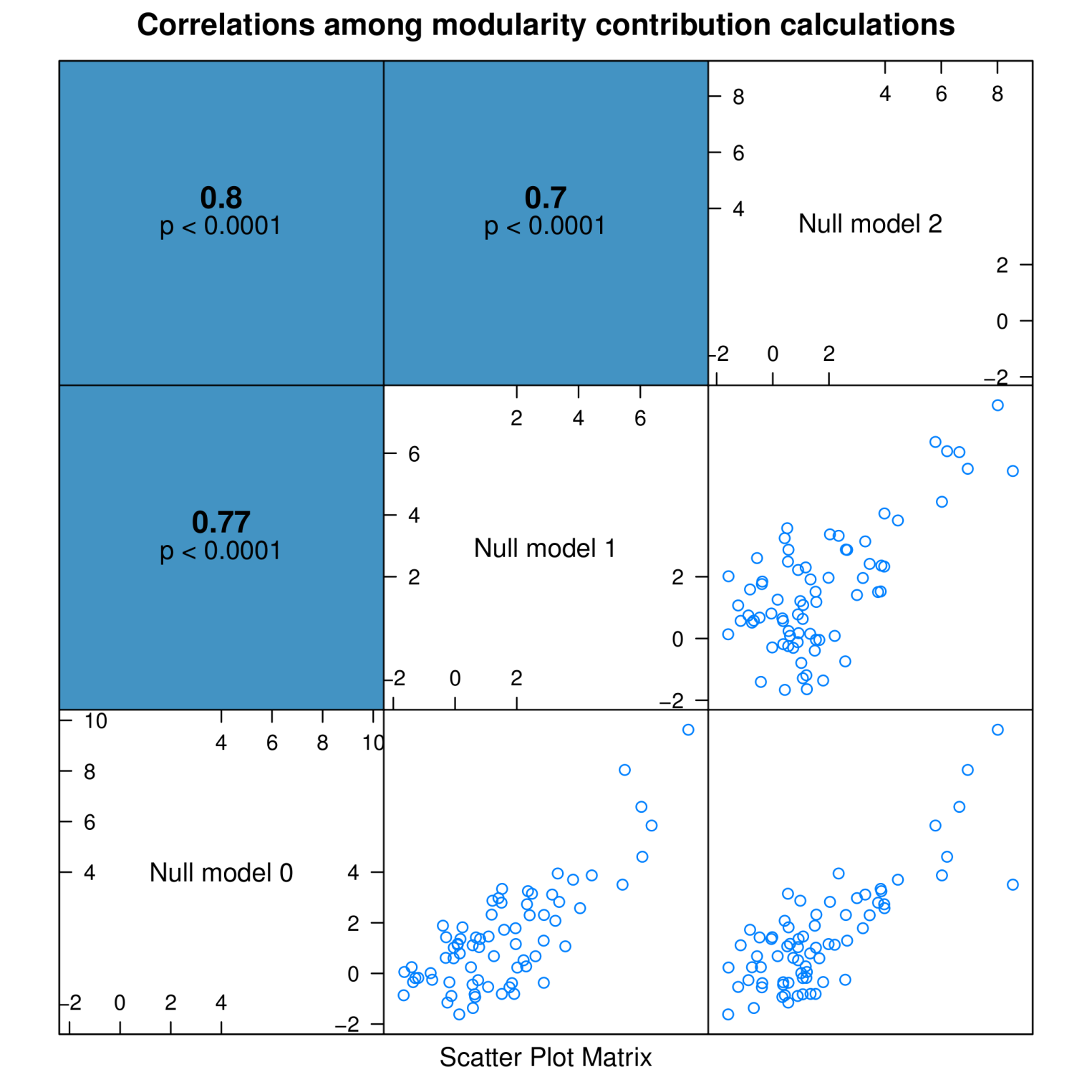


**Figure S2:** Correlations among estimates of host species contributions to modularity, using three different null models. *Null model 0* is the most liberal, allowing cells in the interaction matrix to be filled randomly (e.g. only constraining the total number of interactions). *Null model 1* constrains the probability of an interaction between host and parasite by the number of hosts each parasite infests (e.g. column totals in the host-parasite matrix are constrained). *Null model 2* determines occurrence probabilities according to equation S1, which incorporates information on both row and column totals of the host-parasite matrix.


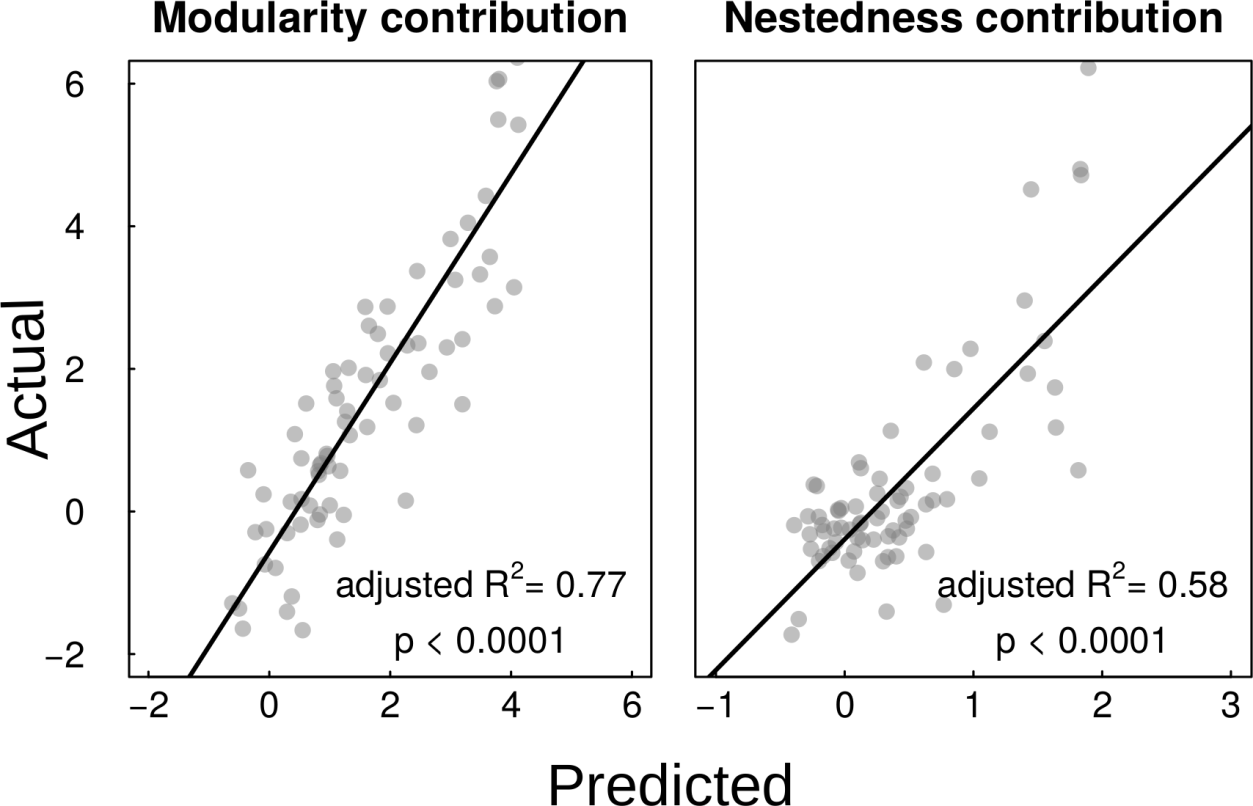


**Figure S3:** Predictive accuracy of the boosted regression models built using host traits (Figure 1 in the main text) to predict the nestedness and modularity contributions of each host species in the fish-parasite network.


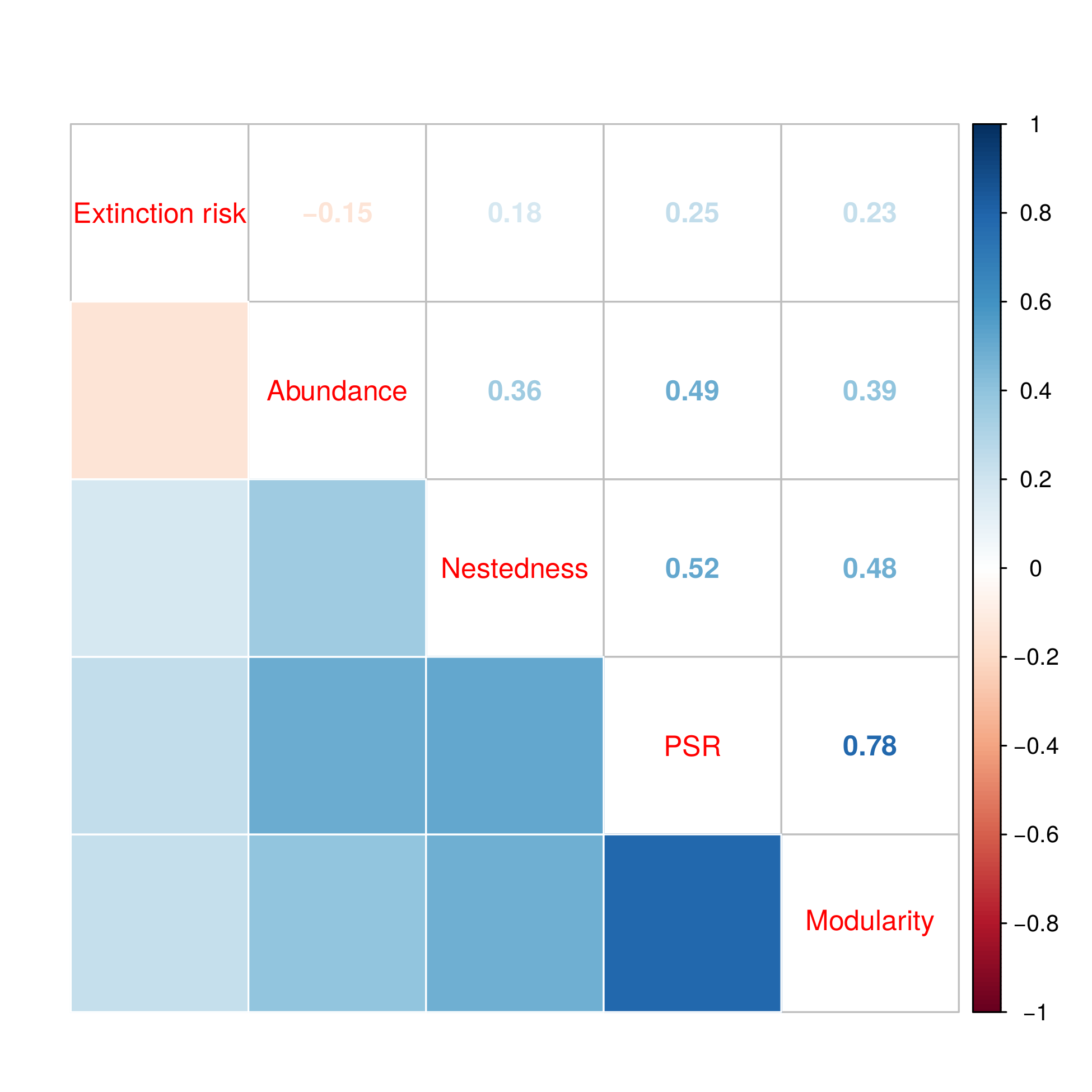


**Figure S4**: Rank correlation matrix depicting the relationship between simulated extinction scenario rankings. High positive values, such as between modularity and parasite species richness (PSR), indicate a large degree of concordance in the ordering of species to be removed (i.e., species with high modularity contribution values also had high parasite richness values). Removal by abundance is included, though this scenario wasn't examined in the main text (but see Figure S5).


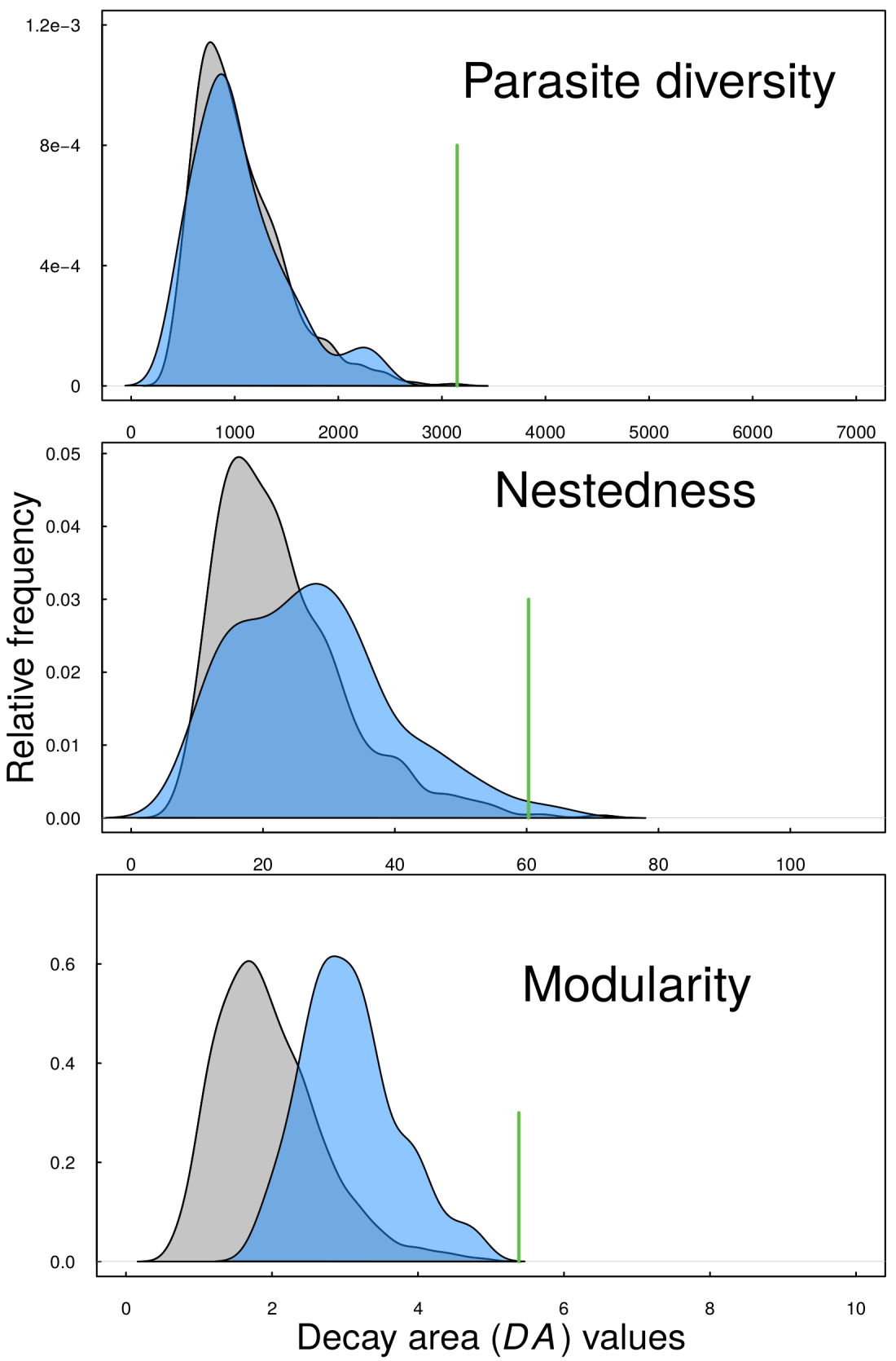


**Figure S5**: Decay area (*DA*) values for two extinction simulations not included in the main text; removal based on status (native or non-native; blue curve) and based on abundance (green line). Removal by status could result in a large number of trajectories, as there are numerous ways to order hosts based on a binary classification. The distribution of AUC values for 100 simulated trajectories where native hosts are lost first are depicted in blue, while the distribution of 1000 simulated random trajectories is provided in grey.


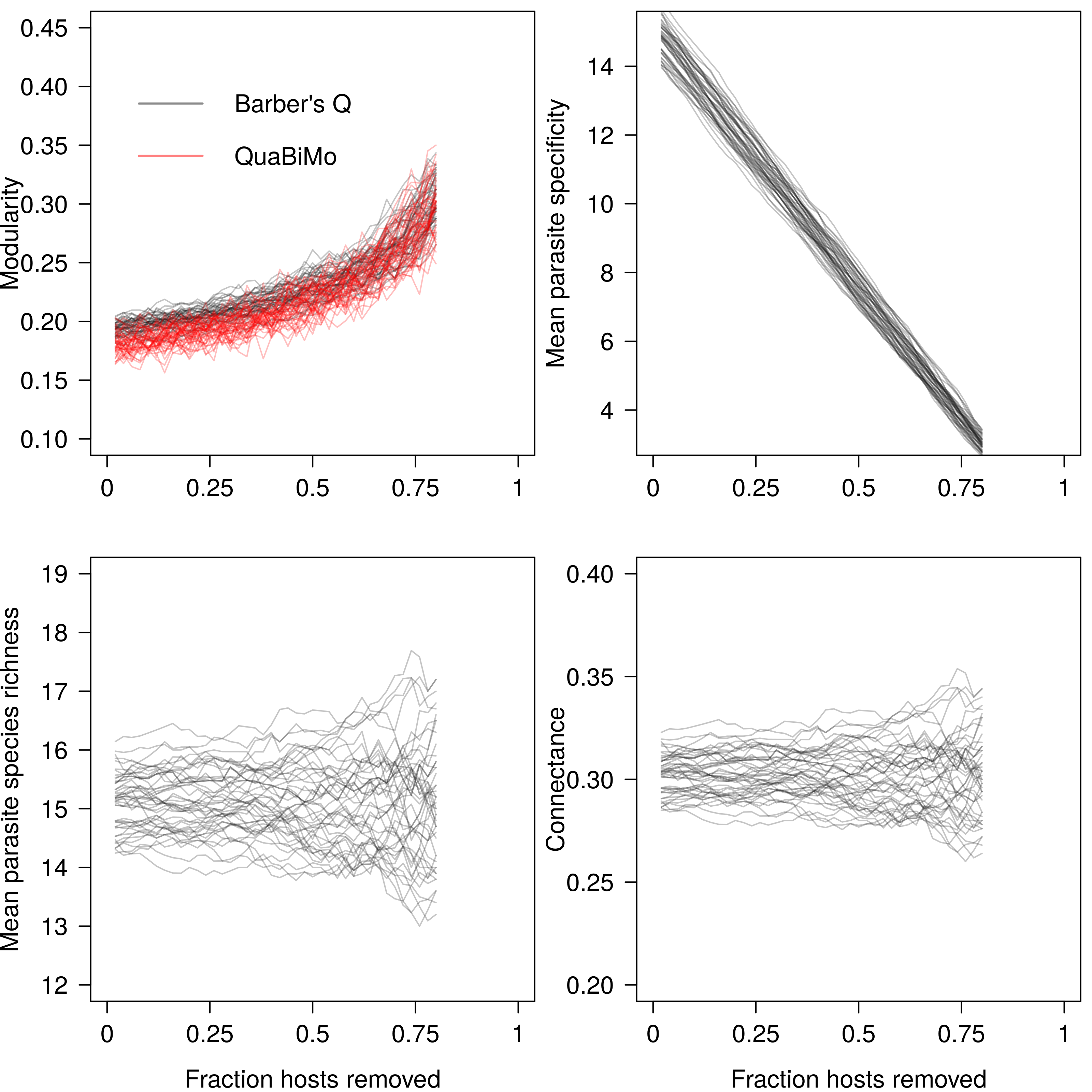


**Figure S6:** Simulated removal of host species from 45 randomly generated bipartite networks. Host species were chosen at random, and modularity is calculated using both Barber's *Q* (Barber 2007), and the QuaBiMo algorithm (Dormann and Straub 2013). Mean parasite specificity is the mean number of hosts parasitized, and was consistently reduced with host removal. No clear relationship was found between random host removal and parasite species richness or connectance.


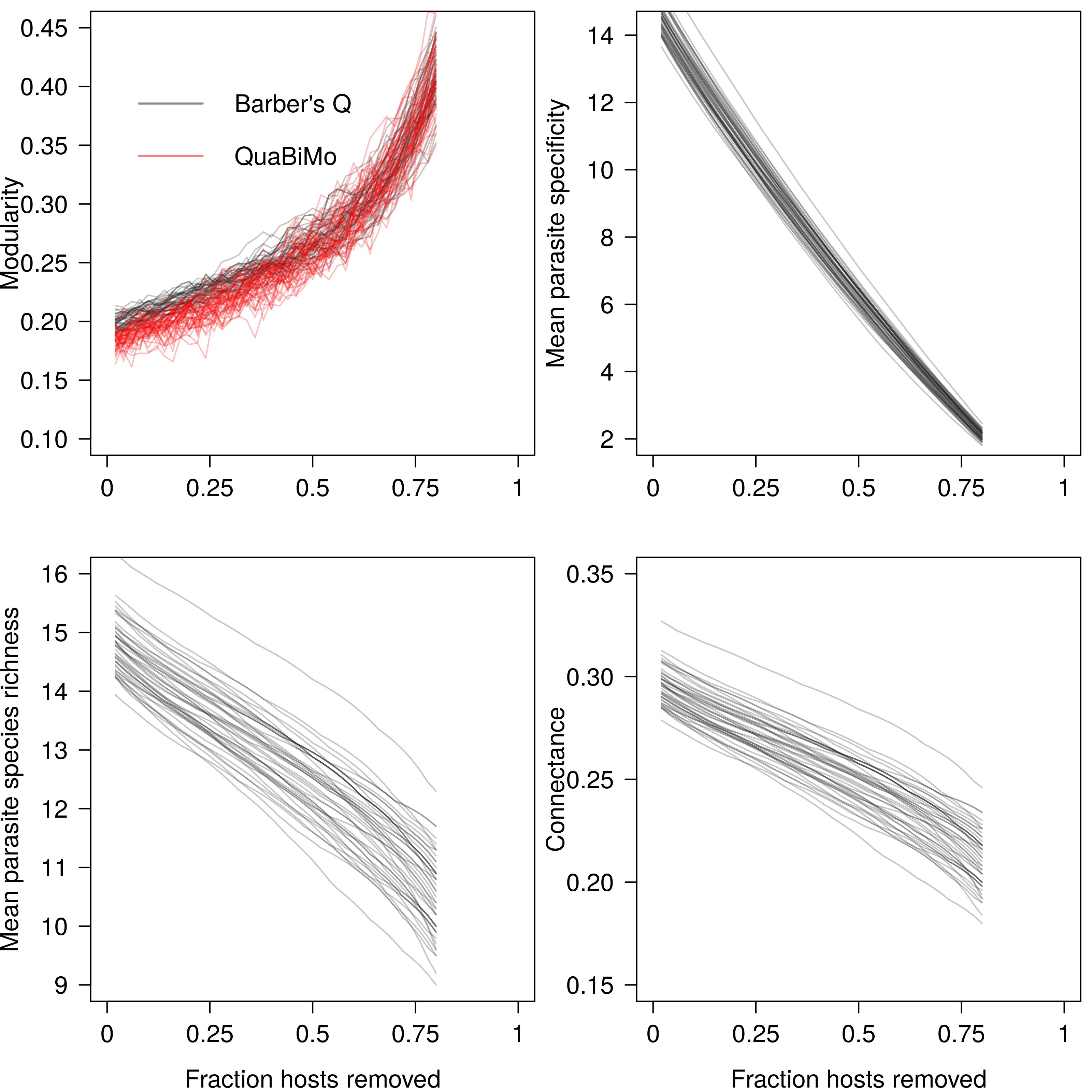


**Figure S7:** Simulated removal of host species from 45 randomly generated bipartite networks. Host species were chosen based on their parasite species richness, with parasite-rich hosts removed first. Modularity is calculated using both Barber's *Q* (Barber 2007), and the QuaBiMo algorithm (Dormann and Straub 2013). Mean parasite richness and connectance decreased with the removal of highly connected hosts, but modularity exhibited a similar pattern when hosts were removed at random, suggesting that the changes to interaction patterns do not drive the increase in modularity with host removal.
